# Supplementary material for: Molecular Evolution of Attachment Glycoprotein (G) and Fusion Protein (F) Genes of Respiratory Syncytial Virus ON1 and BA9 Strains in Xiamen, China
Source: Microbiol Spectr. 2022 Mar 21;10(2):e02083-21. doi: 10.1128/spectrum.02083-21 (PMC9045328; doi:10.1128/spectrum.02083-21)
Supplement: SUPPLEMENTAL FILE 1 — Supplemental material. Download SPECTRUM02083-21_Supp_1_seq1.pdf, PDF file, 0.7 MB [file spectrum02083-21_supp_1_seq1.pdf]

Table S1. Primers and probes for qRT-PCR, amplication and sequencing.

Primer/probe	Sequence (5'-3')	Target gene
qRT-PCR		
RSVA-N-F	gctcttagcaaagtcaagttgaatga	
RSVA-N-R	tgctccgttggatggtgtatt	RSV A/B-N
RSVA-N-P	HEX-acactcaacaaagatcaacttctgtcatccagc-BHQ1	
RSVB -N-F	gatggctcttagcaaagtcaagttaa	
RSVB-N-R	tgtcaatattatctcctgtactacgttgaa	
RSVB-N-P	6-FAM-tgatacattaaataaggatcagctgctgtcatcca-BHQ1	
Amplication /Sequencing		
RSVA-F-F1	ctcaagtctccacaacatccga	RSV A-F
RSV F-R2	gcaatgacctcgaatttcaaatt	
RSVB-F-F1	cacaaacacccacagcatccga	RSV B-F
RSV F-R2	gcaatgacctcgaatttcaaatt	
RSVA/B-G-F	gggcaaatgcaaacatgtcc	RSV A/B-G
RSVA/B-G-R	gcaactccatkgttatttgcc	

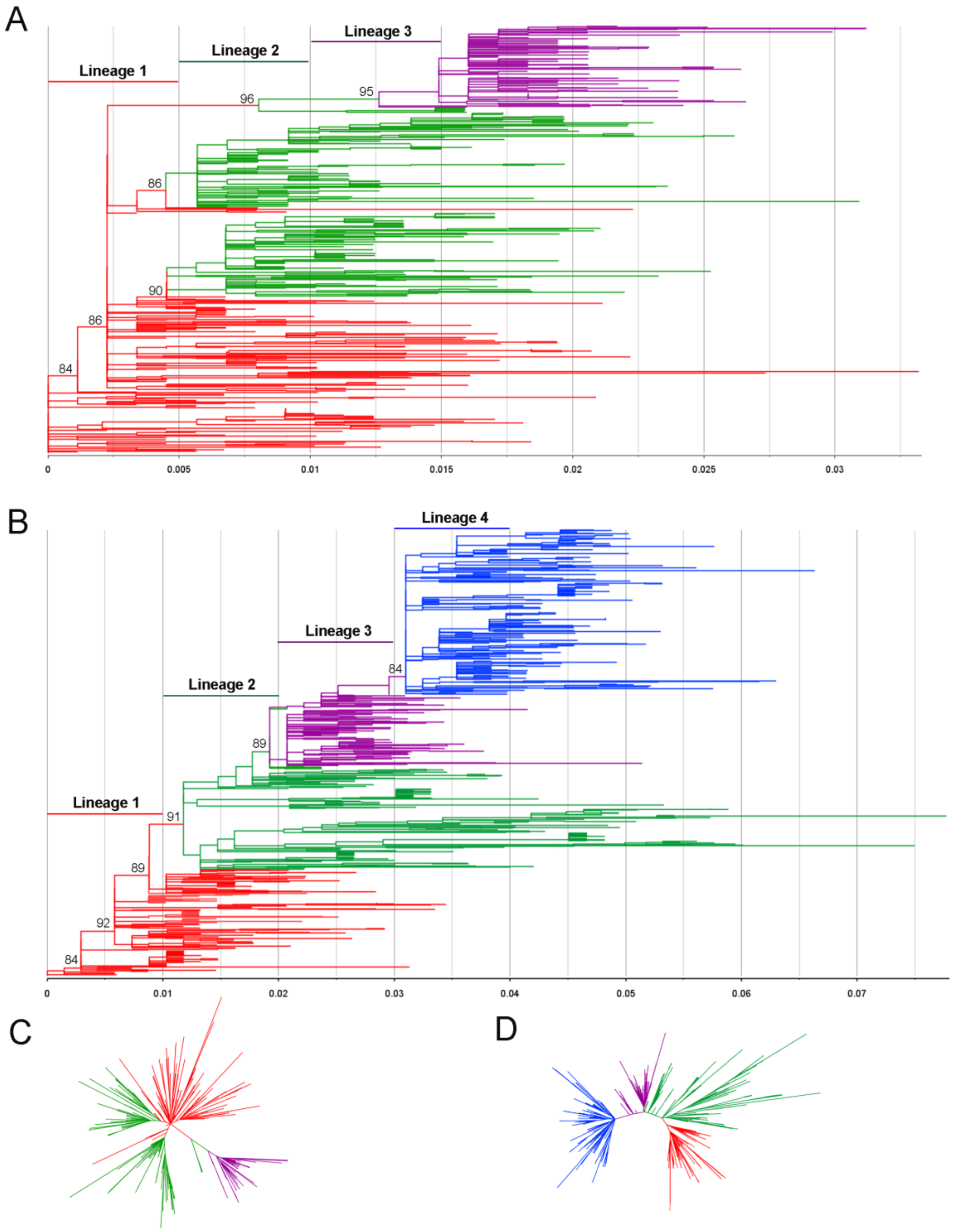

Figure S1. Lineages identified in the ML trees of RSV G. ML trees are displayed in rectangular format for the G protein ectodomain of Xiamen RSV A (A) and B (B) strains and global circulating strains during 2010-2019. Branches of different lineages are highlighted by different colors (red for Lineage 1; green for Lineage 2; purple for Lineage 3; blue for Lineage 4). Each lineage name is listed above the colored line. A cut-off of 0.005 patristic distance was set to define lineages for RSV A and 0.01 for RSV B.

Ultrafast bootstrap values of each ancestral lineage node as statistical support are displayed on the trees. The radial trees of RSV A (C) and B (D) strains are showed under the graph.

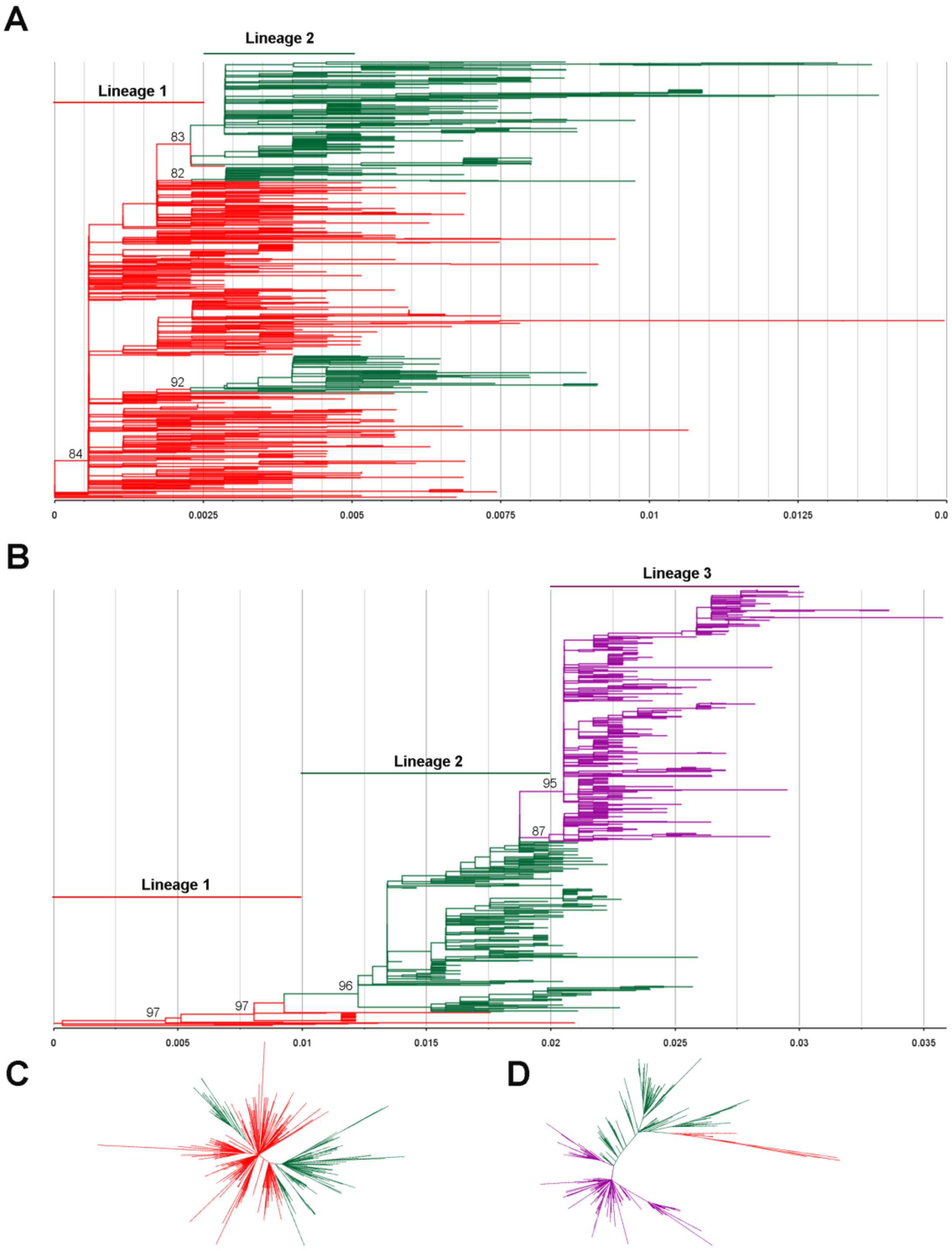

Figure S2. Lineages identified in the ML trees of RSV F. ML trees are displayed in rectangular format for F protein of Xiamen RSV A (A) and B (B) strains and global

circulating strains during 2010-2019. Branches of different lineages are highlighted by different colors (red for Lineage 1; green for Lineage 2; purple for Lineage 3). Each lineage name is listed above the colored line. A cut-off of 0.0025 patristic distance was set to define lineages for RSV A and 0.01 for RSV B. Ultrafast bootstrap values of each ancestral lineage node as statistical support are displayed on the trees. The radial trees of RSV A (C) and B (D) strains are showed under the graph.

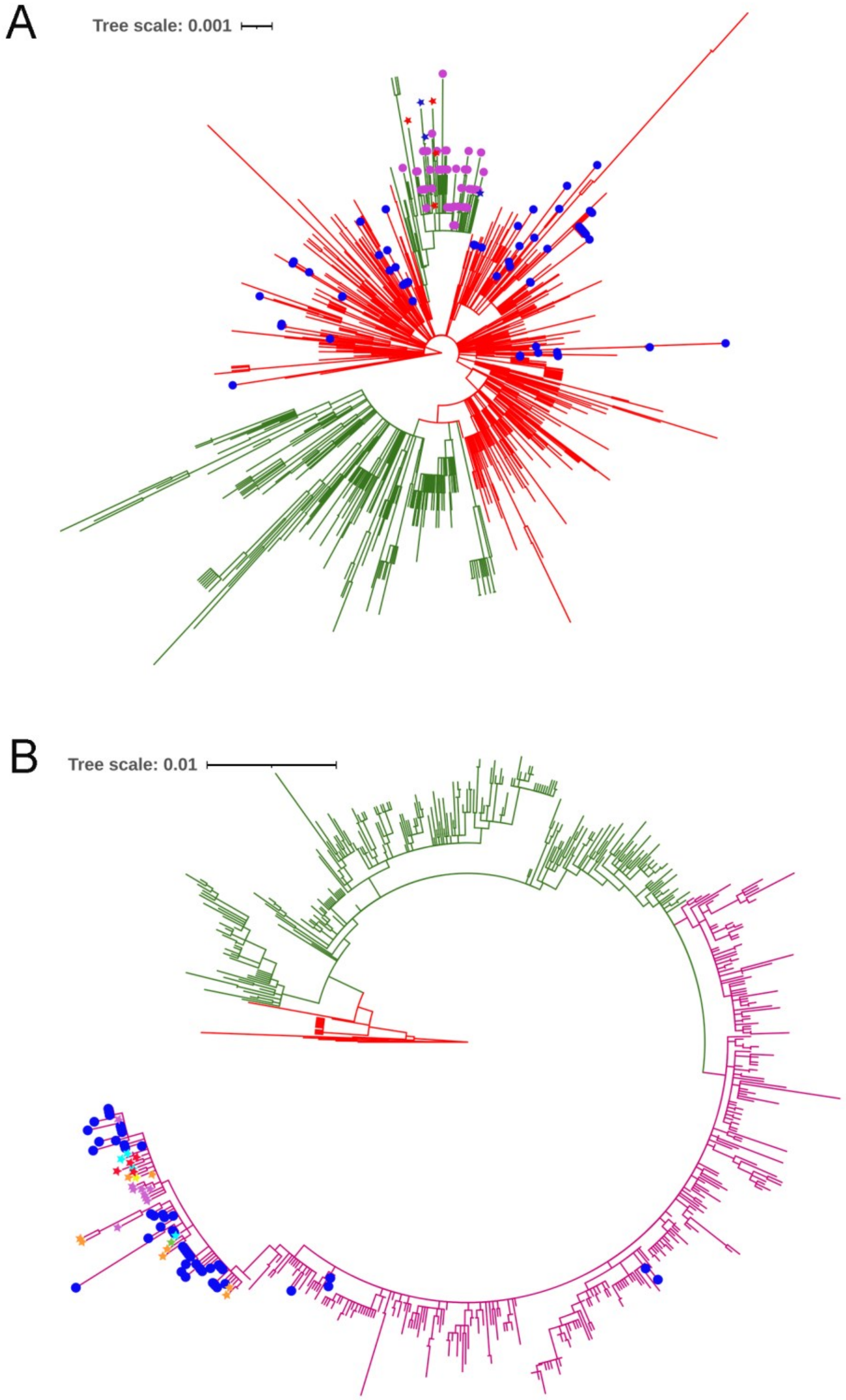

Figure S3. ML trees for F protein of Xiamen RSV A and B strains and global circulating strains (2010-2019). ML trees were constructed using the ML method with IQ-TREE and displayed in circular format. Branches of different lineages are highlighted by different colors (red for Lineage 1; green for Lineage 2 and purple for Lineage 3). Xiamen strains obtained in this study are marked by colored dots. American and Russian

strains are labelled by red and blue stars respectively in Lineage 2 in the ML tree for RSV A strains. Similarly, Australian (purple stars), Japanese (cyan stars), American (yellow stars), Switzerland (green stars), Russian (orange stars) and Chinese (red stars) strains are labelled by colored stars in Lineage 3 in the ML tree for RSV B strains.
